# Psychometric assessments of Persian translations of three measures of conspiracist beliefs

**DOI:** 10.1371/journal.pone.0215202

**Published:** 2019-04-18

**Authors:** Mohammad Atari, Reza Afhami, Viren Swami

**Affiliations:** 1 Department of Psychology, University of Southern California, Los Angeles, United States of America; 2 Department of Art Studies, Tarbiat Modares University, Tehran, Iran; 3 School of Psychology and Sport Science, Anglia Ruskin University, Cambridge, United Kingdom; 4 Center for Psychological Medicine, Perdana University, Serdang, Malaysia; University of Lleida, SPAIN

## Abstract

Several self-report measures of conspiracist beliefs have been developed in Western populations, but examination of their psychometric properties outside Europe and North America is limited. This study aimed to examine the psychometric properties of three widely-used measures of conspiracist beliefs in Iran. We translated the Belief in Conspiracy Theory Inventory (BCTI), Conspiracy Mentality Questionnaire (CMQ), and Generic Conspiracist Belief Scale (GCBS) into Persian. Factorial validity was examined using principal-axis factor analysis in a community sample from Tehran, Iran (*N* = 544). Further, the relationships between scores on these measures and hypothesized antecedents (i.e., education, schizotypal personality, information processing style, superstitious beliefs, religiosity, and political orientation) were examined. Overall, we failed to find support for the parent factor structures of two of the three scales (BCTI and GCBS) and evidence of construct validity for all three scales was limited. These results highlight the necessity of further psychometric work on existing measures of conspiracy theories in diverse culturo-linguistic groups and the development of context-specific measures of conspiracist beliefs.

## Introduction

Some people believe immunization does not serve its intended purpose, global warming is a hoax, humans never landed on moon, and the United States (U.S.) government was involved in the 9/11 terrorist attacks. Such beliefs are commonly referred to as *conspiracy theories*, broadly defined as a subset of false narratives in which the ultimate cause of an event is believed to be due to a malevolent plot by multiple agents secretly working together [1–2]. A large proportion of the population of some countries share such beliefs; for example, Oliver and Wood [3] reported that approximately 55% of American adults in a nationally representative survey in the U.S. agreed with at least one of seven conspiracy theories they were presented with. Indeed, conspiracy theories spread rapidly across socio-political spectra despite strong contra-indicatory evidence [4–6]. In the past two decades, there has been increasing interest in psychological predictors and outcomes of endorsing conspiracy theories.

Douglas, Sutton, and Cichocka [7] suggested that belief in conspiracy theories is primarily driven by motives that can be characterized as epistemic (i.e., understanding one’s environment), existential (i.e., being safe and in control of one’s surroundings), and social (i.e., maintaining a positive image of one’s self and social group). In addition, belief in conspiracy theories have been shown to be associated with individual differences in cognitive [8–10], psychosocial [11], and personality [12] factors. Specifically, conspiracy beliefs are correlated with lower levels of analytic thinking [8,10] and lower levels of education [13–14]. In addition, feelings uncertainty may lead individuals to endorse conspiracy theories [15], so as to restore feelings of control. Although conspiracist beliefs show equivocal correlations with normative personality traits, such as the Big Five (see [12]), they have been found to be strongly associated with schizotypal personality facets [8,16]. In addition, religiosity and political extremism have been implicated to varying extents in beliefs in conspiracy theories, although relationships have tended to be weak and equivocal in direction [13,17–18].

The aforementioned literature has relied on self-reports of conspiracist beliefs that can be categorized into two broad streams (for a review, see [19]): measures of a range of conspiracy theories (i.e., instruments that make reference to a range of real-world conspiracy theories) and measures of generic conspiracy ideation (i.e., instruments that do not make reference to specific conspiracy theories). Among measures in the former stream, the Belief in Conspiracy Theory Inventory (BCTI) [12] is perhaps the most widely-used. In the parent study, Swami et al. [12] subjected 15 items to a principal-axis factor analysis (PAFA) and reported that all but one of the items loaded onto a primary general factor. They, therefore, computed a total BCTI score as the mean of the 14 remaining items, a method that has also been used in one subsequent study [2]. In another study [20], an additional item was added to the list of 14 items and a total score was computed, and recent studies have mostly used this adapted 15-item version of the BCTI [11]. The most recent psychometric work on the BCTI, conducted with U.S. samples [19], showed satisfactory psychometric properties for the BCTI, including a one-dimensional factor structure via both PAFA and confirmatory factor analysis (CFA), as well as adequate patterns of convergent validity.

In the second stream (i.e., general conspiracist beliefs), two measures have been subjected to rigorous psychometric investigation: the Conspiracy Mentality Questionnaire (CMQ) [21] and the Generic Conspiracist Belief Scale (GCBS) [22]. Two different versions of the CMQ are available: a 12-item version [23] and a shorter 5-item version [21]. The first version has been subjected to CFA, which showed a one-factor solution to have acceptable fit to the data, but CFA is an imperfect factor analytic strategy for a newly developed scale without previous exploratory analysis. Conversely, Bruder and colleagues [21] subjected the five-item version of the measure to PAFA and extracted one general factor termed “conspiracist mentality.” Furthermore, multi-group CFA showed that the one-dimensional model had adequate fit in German- and English-speaking samples, but fit indices for a Turkish-speaking sample were relatively poor. On the other hand, the GCBS initially consisted of a pool of 75 items, but was reduced to 59 items following exclusion of negatively-worded items. Results of an exploratory factor analysis suggested a five-factor solution for the remaining items. In a second study, Brotherton et al. [22] identified 15 “representative” items and reported that CFA showed a five-factor model to have acceptable fit to the data. Both the CMQ and GCBS have been translated into a handful of languages outside their parent studies, but sustained evaluations of their psychometric properties are limited (for a review, see [19]).

In a large study of U.S. participants, Swami et al. [19] identified major problems with the factor structures of both the CMQ and GCBS. In terms of the CMQ, PAFA supported a one-factor structure, but CFA indicated that the one-factor model had poor fit to the data. Swami et al. [19] also highlighted additional issues with this measure, including poor construct validity (i.e., it is debatable whether the CMQ in fact measures conspiracist beliefs as opposed to factual beliefs about the state of the world). On the other hand, PAFA with the GCBS supported a two-factor solution consisting general and extraterrestrial conspiracist items, respectively [19]. However, CFA indicated that this two-factor model had poor fit to the data, as did the parent five-factor and one-factor models. As Swami et al. [19] noted, inherent problems with the analytic strategy in the parent study reporting on the GCBS may have introduced problems with item content, functioning, and structure. These authors went on to recommend measurement refinement for future research using the GCBS, as well as the BCTI (they also suggested that future studies should not use the CMQ given problems with its construct validity).

In addition to the limitations noted above, there is a severe lack of cross-cultural data and evidence supporting the reliability and validity of these measures, particularly in non-Western cultures. Indeed, there has been very little work on conspiracy theories in non-Western cultures, which impedes cross-cultural comparisons and international collaboration in this growing line of research. Even where data from non-English speaking samples is obtained, it remains heavily reliant on European samples (e.g., [9]) and/or does not adequately consider psychometric issues (e.g., [24]). The former is problematic because, as Henrich, Heine, and Norenzayan [25] point out, limiting participants to people from Western, educated, industrialized, rich, and democratic (WEIRD) societies, and particularly U.S. college students, has practical consequences for our understanding of the psychological constructs being investigated. Likewise, the latter issue is problematic because scholars may be erroneously assuming that latent dimensionality uncovered in one population is suitable for another, without adequate testing of such an assumption.

### The present study

In view of the issues discussed above, the present study aimed to investigate the psychometric properties of widely-used measures of conspiracy ideation in Iran, a non-Western country with an interesting geopolitical position *vis-à-vis* conspiracist beliefs. Iran is a Muslim-majority country in Middle East, categorized as a high-development country in the latest United Nations Development Programme [26], and where Persian (or Farsi) is the official language. Although the study of conspiracy theories in Iran is limited, one early study by Zonis and Joseph [27] suggested that rapid social and cultural change in Iran meant that Iranians frequently resorted to conspiracy theories to account for new and contested realities. In their view, national and socio-cultural crises induced a “regression in mental processes” and facilitated the emergence of conspiracy theories [27]. These authors also speculated that child-rearing practices (e.g., the loss of dominant father figures) and the secrecy of sexuality in Iran heightened paranoid-like ideation, which in turn enhanced a tendency to believe in conspiracy theories. Although some of this commentary may now be antiquated, it is also apparent that conspiracy theories enjoy mainstream audiences in Iran specifically and the Middle East more broadly [28]. Indeed, recent work has suggested that belief in conspiracy theories, particularly with regards to international relations, Zionism and the creation of Israel, Western military intervention in the Middle East, and oil pricing, may be particularly charged in the Iranian context [29–30]. Thus, Iran is an interesting cultural setting for scientific investigations of conspiracy theories, but such work will need to be predicated on appropriate psychometric measures of conspiracist beliefs.

As such, we undertook psychometric evaluations of Persian versions of the BCTI, CMQ, and GCBS (as the shortest and best-performing instruments described above; see Swami et al. [19] for a review) in a relatively large community sample from Iran. Based on the findings of Swami et al. [19], we expected that the BCTI and CMQ would reduce to one-factor models, whereas the Persian GCBS would reduce to a two-factor model. In addition, we also conducted an assessment of the construct validity of these measures by investigating associations with education, schizotypal personality, information processing style, superstitious beliefs, religiosity, and political orientation. Each of these constructs was selected as being appropriate for the estimation of construct validity based on established associations with conspiracist beliefs in previous studies (see above). Evidence of construct validity would be provided through significant and moderate-to-strong positive correlations between scores on the adapted measures of conspiracist ideation and schizotypal personality, intuitive information processing style, superstitious beliefs, rightist political orientation, and religiosity. On the other hand, we hypothesized the scores on the aforementioned measures of conspiracy beliefs would be negatively and moderately-to-strongly correlated with education and analytical information processing style. More broadly, by including these additional measures, we were able to examine the extent to which the associations between conspiracist beliefs and proposed correlates would be replicated in a new cultural context.

## Methods

### Participants

The sample consisted of 544 individuals (52.6% male, 37.5% female, 9.9% preferred not to report). All participants were recruited from the general population from public places in Tehran, Iran. Participants ranged in age from 15 to 75 (*M* = 32.5, *SD* = 9.8 years). Two-hundred and thirty participants (42.3%) were unmarried and 299 participants (55%) were married. Fifteen participants chose not to disclose their marital status. In terms of highest education qualification, 31 participants (5.7%) reported some high school education, 104 participants (19.1%) reported having a high school diploma, 121 participants (22.2%) reported an associate’s degree, 191 participants (35.1%) reported a bachelor’s degree, 81 participants (14.9%) reported a postgraduate degree, and 8 participants (1.5%) reported a doctoral degree. Eight participants did not report their highest educational qualification.

### Measures

#### Belief in Conspiracy Theory Inventory (BCTI)

The version of the BCTI that we used was the 15-item, adapted version. This includes 14 items from the parent study [12] and an additional item added in a subsequent study [20]. The factor structure of this adapted version of the BCTI has been investigated is U.S. adults using PAFA and CFA by Swami et al. [19], who found a one-factor solution in their exploratory analysis. Further, these authors reported acceptable fit indices for a one-factor model in their CFA. In the present study, all items were rated on a 9-point scale, ranging from 1 (*Completely false*) to 9 (*Completely true*). Higher scores on this scale reflect greater endorsement of a range of real-world conspiracy theories.

#### Conspiracy Mentality Questionnaire (CMQ)

All participants completed the 5-item version of the CMQ. Bruder et al. [21] reported that the 5-item CMQ had a one-dimensional structure using EFA and that the fit was adequate in German- and English-speaking samples using CFA. More recently, Swami et al. [19] found fit indices of the one-dimensional model of CMQ to be poor in a U.S. sample. Although there are criticisms with regard to CMQ’s response option [19], we maintained and translated its original format in the current study. All participants were asked to rate their certainty on an 11-point scale ranging from 0% (*Certainly not*) to 100% (*Certain*). The 11-point scale was coded from 0 to 10. Higher scores on this scale reflect greater generic conspiracist ideation.

#### Generic Conspiracist Belief Scale (GCBS)

We used the 15-item version of the GCBS in the current study [22]. The 15 items were selected by Brotherton and colleagues [22] from a larger pool of items to be representative of the five-factor solution reported in the parent study. The authors reported that a five-factor solution had adequate fit using CFA and that this model also had better fit than a one-factor solution with all items. In their psychometric evaluation, however, Swami et al. [19] found a two-factor solution using PAFA, with one 6-item factor (general conspiracist beliefs) and another 4-item factor (extraterrestrial conspiracist beliefs). However, CFA indicated poor fit to the data for the two-factor model, as well as the parent five-factor and one-factor models. In the current study, items were rated on a 5-point scale, ranging from 1 (*Definitely not true*) to 5 (*Definitely true*). Higher scores on this scale reflect greater generic conspiracist ideation.

#### Schizotypal Personality Questionnaire-Brief (SPQ-B)

The SPQ-B is a 22-item self-report instrument derived from the 74-item SPQ, designed according to *Diagnostic and Statistical Manual of Mental Disorders-III-Revised* (*DSM-III-R*) diagnostic criteria for Schizotypal Personality Disorder [31–32]. Each positive item indicates the presence of an SPD symptom. Items were created to measure three dimensions of SPD: 8 items for cognitive-perceptual (i.e., ideas of reference, odd beliefs, magical thinking, unusual perceptual experiences, suspiciousness and paranoid ideation), 8 items for interpersonal (i.e., suspiciousness, inappropriate or constricted affect, lack of close friends and excessive anxiety), and 6 items for disorganization (i.e., odd thinking/speech and odd or eccentric behavior/appearance) [33]. The Persian translation of the SPQ-B has been shown to result in relatively low factor-level internal consistencies [34], so more recent studies have used the total score of the Persian SPQ-B [35]. In the current sample, internal consistency coefficient for the total score was .71.

#### Iranian Superstitious Beliefs Questionnaire (ISBQ)

All participants completed a measure of superstitious beliefs and magical thinking specific to Iranian culture [36]. The ISBQ is a self-report measure of culture-specific supernatural thinking with 10 questions (stem: *Do you believe…*) rated on a 4-point scale from 1 (*I do not believe at all*) to 4 (*I strongly believe so*). The ISBQ has a two-factor structure: superstitious beliefs (5 items) and magical thinking (5 items). In the current study, the internal consistency coefficients were .80 and .74, for superstitious beliefs and magical thinking, respectively.

#### Self-Rating of Religiosity (SRR)

All participants completed the SRR [37] on an 11-point scale ranging from *0* (indicating no religiosity) to *10* (indicating high level of religiosity). The SRR is a single-item measure of religiosity. Single-item measures are generally limiting in terms of breadth, but can provide important data especially in community samples, such as the current study. The Persian translation of the SRR has been shown to have good convergent validity with longer religiosity measures in Iran and has high test-retest reliability over a three-week period of time [38].

#### Political orientation

Participants rated their affiliation with the rightist political party in Iran as opposed to the leftist party along a 7-point scale ranging from 1 (*Very leftist*) to 7 (*Very rightist*). Another item asked participants to rate their political conservatism on a scale ranging from 1 (*Very liberal*) to 7 (*Very conservative*). We averaged these two items in order to achieve a political orientation score, where higher scores indicated more rightist political orientation. Previous studies have used a similar method for assessment of political orientation in other cultures [39]. The internal consistency coefficient of these two items was relatively low in the current sample (Cronbach’s α = .43), but two-item measures commonly show very low internal consistencies.

#### Rational-Experiential Inventory (REI)

In the present study, we used the REI [40] to measure participants’ information processing style. This 40-item self-report measure examines information processing mode preferences, with answers loading on two independent dimensions: intuitive-experiential (REI-E: 20 items) and analytical-rational (REI-R: 20 items). Participants rated each item on a 5-point Likert-type scale, from 1 (*Completely false*) to 5 (*Completely true*). Higher scores indicate stronger self-reported preference to use the respective thinking style (i.e., intuitive-experiential vs. analytical-rational). Rezaei [41] subjected the Persian translation of the 40-item REI to exploratory factor analysis in an Iranian sample and retained 22 items of the REI-40. Therefore, we used the 22-item Persian REI, but further discarded items with low inter-item and item-total correlations, which resulted in 8 items for intuitive-experiential subscale (Cronbach’s α = .80) and 6 items for analytical-rational subscale (Cronbach’s α = .82).

#### Demographics

Participants provided their demographic details consisting of sex, age, current marital status, and highest educational qualifications.

### Procedure

All procedures were approved in the ethics committee of the University of Tehran, consistent with the principles expressed in the Declaration of Helsinki. Once ethics approval was obtained from the relevant university ethics committee, we prepared Persian translations of the BCTI, CMQ, and GCBS from the parent English versions using the standard back-translation technique [42]. We, then, administered the translations to 30 students at the University of Tehran and asked for feedback regarding readability and clarity of items. Some issues were raised. Smaller linguistic and semantic issues in translation were resolved by an independent bilingual translator. One major issue that emerged was unfamiliarity with some item content on the BCTI (e.g., Martin Luther King or Area 51 in the U.S.). In a further focus group with a number of graduate-level students, we attempted to clarify such items. To do so, we added brief information about the unfamiliar items in the BCTI in item stems (see Appendix 1). For the main study. Two research assistants approached potential participants and invited them to take part in a psychological research about “values and beliefs.” The order of presentation of all scales in the study was pre-randomized. All participants completed a set of paper-and-pencil measures after giving oral informed consent to take part in the study. Participants were not compensated, but were provided with debriefing information upon completion of the questionnaire.

### Statistical analysis

Following standard guidelines [43], items of BCTI, CMQ, and GCBS were submitted to PAFA if they passed standard criteria. For the BCTI and CMQ, we used quartimax rotations because of the expectation of a single factor. For the GCBS, however, we used a varimax rotation because we expected an inter-correlated, multidimensional structure [44–45]. The number of factors to be extracted was determined by factor eigenvalues (λ) above 1.0 (the EGV1 criterion), examination of the scree-plot, and where more than one factor was identified through rotation, the results of parallel analysis [46]. We specifically used O’Connor’s [47] syntax to conduct parallel analysis, which was used because scree-plot inspection and the EGV1 criterion are known to lead to factor over-retention [47–49]. Parallel analysis creates random datasets with the same number of cases and variables as the actual dataset. Factors in the actual data are only retained if their eigenvalues are greater than the mean of eigenvalues (with 95% CI) from the random data [46]. Factor loadings were interpreted using Tabachnick and Fidell’s [50] recommendations and highly cross-loading items were discarded.

Internal consistency coefficients of scales and subscales were computed using Cronbach’s α. Although Nunnally [51] is widely interpreted as suggesting a cut-off of .70, this is in fact a “myth” [52]. In fact, Nunnaly [51] advocated a more conservative cut-off of .80, which was applied for newly adapted measures (i.e., BCTI, CMQ, and GCBS) in the present study. To assess convergent validity, we computed bivariate correlations between all study variables. According to Lipsey and Wilson [53, 54], correlations of .10 are considered small, correlations of .25 are considered medium, and correlations of .40 are considered large. We used independent-samples *t*-tests to examine between-group differences between women and men. Cohen’s *d* [54] was computed as a measure of effect size. Effects are considered as large if differences are greater than 0.80, moderate if differences are between 0.50 and 0.80, and small if differences are between 0.20 and 0.50. Although we report *p* < .05 as our statistical significance level [55], we flag correlations where *p* < .005. Interpretations of convergent validity were based on the magnitude of the effects, rather than solely on statistical significance. In case of multiple comparisons, we applied Bonferroni correction to compensate for Type I error.

## Results

### Factor analyses

#### BCTI

The BCTI items were examined for normality of distribution and were found to be lower than limits; these items were, therefore, appropriate for factor analysis. The participant-item ratio was 36.3, much larger than the recommended minimums which typically range between 5 and 20 [48]. Therefore, the sample size was adequate for factor analysis. The size of Kaiser-Meyer-Olkin (KMO) measure of sampling adequacy suggested that the BCTI items had adequate common variance for factor analysis (KMO = .89). Bartlett’s test of sphericity, χ^2^(105) = 2589.10, *p <* .001, indicated that the correlation matrix was factorable. The results of the PAFA revealed three factors with λs larger than one (5.54, 1.44, and 1.21). However, inspection of the scree plot suggested one primary factor and a steep cut-off to the secondary factor. The results of parallel analysis showed that the first two eigenvalues (95% CI means = 1.38 and 1.29) for the random data were smaller than the real data, whereas the third permuted eigenvalue (95% CI mean = 1.23) was larger than the third eigenvalue for the real data (λ = 1.21). These findings suggest that two factors should be extracted, although it may be noted that all 15 items had good loadings on the primary factor (see Table 1). The two extracted factors cumulatively explained 46.6% of the total item variance. Only three items loaded on the second factor (loadings > .45). Based on Tabachnick and Fidell’s [50] recommendations, we eliminated cross-loading items (Items #1, #2, and #3), leaving 12 items adequately loading onto the first factor (see Table 1; Cronbach’s α = .85).

**Table 1:** Descriptive statistics and Factor Loadings for Belief in Conspiracy Theory Inventory (BCTI) Items.

Item	*M (SD)*	Loadings	
		F1	F2
8. The US government allowed the 9/11 attacks to take place so that it would have an excuse to achieve foreign (e.g., wars in Afghanistan and Iraq) and domestic (e.g., attacks on civil liberties) goals that had been determined prior to the attacks.	5.41 (2.40)	.63	.02
9. The assassination of John F. Kennedy was not committed by the lone gunman, Lee Harvey Oswald, but was rather a detailed, organized conspiracy to kill the President.	5.42 (2.32)	.62	.03
11. Princess Diana’s death was not an accident, but rather an organized assassination by members of the British royal family who disliked her.	5.24 (2.28)	.60	-.16
5. The assassination of Martin Luther King, Jr., was the result of an organized conspiracy by US government agencies such as the CIA and FBI.	5.40 (2.22)	.60	.35
10. In July 1947, the US military recovered the wreckage of an alien craft from Roswell, New Mexico, and covered up the fact.	5.07 (2.27)	.60	-.17
3. The US government had foreknowledge about the Japanese attack on Pearl Harbor, but allowed the attack to take place so as to be able to enter the Second World War.*	5.46 (2.26)	.59	.45
6. The Apollo moon landings never happened and were staged in a Hollywood film studio.	5.02 (2.30)	.59	-.03
7. Area 51 in Nevada, US, is a secretive military base that contains hidden alien spacecraft and/or alien bodies.	4.99 (2.26)	.59	-.13
12. The Oklahoma City bombers, Timothy McVeigh and Terry Nichols, did not act alone, but rather received assistance from neo-Nazi groups.	5.07 (2.17)	.59	-.16
4. US agencies intentionally created the AIDS epidemic and administered it to Black and gay men in the 1970s.	5.12 (2.41)	.59	.29
1. A powerful and secretive group, known as the New World Order, are planning to eventually rule the world through an autonomous world government, which would replace sovereign government.*	5.66 (2.45)	.50	.49
13. The Coca Cola company intentionally changed to an inferior formula with the intent of driving up demand for their classic product, later reintroducing it for their financial gain.	5.03 (2.32)	.50	.03
15. Government agencies in the UK are involved in the distribution of illegal drugs to ethnic minorities.	5.20 (2.40)	.44	.01
14. Special interest groups are suppressing, or have suppressed in the past, technologies that could provide energy at reduced cost or reduced pollution output.	5.38 (2.34)	.44	.12
2. SARS (Severe Acute Respiratory Syndrome) was produced under laboratory conditions as a biological weapon.*	5.39 (2.42)	.51	.60

*Note*. F1 = Factor 1; F2 = Factor 2; Items with an asterisk were discarded. The Persian translation is available upon request.

#### CMQ

Tests of normality of distribution showed that the CMQ items were lower than recommended limits. The participant-to-item ratio for CMQ was 108.8, which is larger than the recommended minimums. The size of KMO measure of sampling adequacy, KMO = .81, suggested that the CMQ items had adequate common variance for factor analysis, and Bartlett’s test of sphericity, χ^2^(10) = 1289.37, *p <* .001, indicated that the correlation matrix was factorable. The results of the PAFA revealed a single factor with λ = 3.25, which explained 64.9% of the variance. All 5 items had excellent loadings on this factor (see Table 2). Cronbach’s α for the overall CMQ score, computed as the mean of all 5 items, was .86.

**Table 2:** Descriptive Statistics and Factor loadings for Conspiracy Mentality Questionnaire (CMQ) Items.

Item	*M (SD)*	Loading
2. I think that politicians usually do not tell us the true motives for their decisions.	8.06 (2.21)	.81
1. I think that many very important things happen in the world, which the public is never informed about.	7.80 (2.48)	.79
3. I think that government agencies closely monitor all citizens.	7.13 (2.51)	.73
4. I think that events which superficially seem to lack a connection are often the result of secret activities.	7.21 (2.45)	.71
5. I think that there are secret organizations that greatly influence political decisions.	7.79 (2.29)	.70

*Note*. The Persian translation is available upon request.

#### GCBS

The GCBS items were examined for normality of distribution and were found to be lower than limits. The participant-to-item ratio was 36.3, which is much larger than the recommended minimums. The size of the KMO (.89) and Bartlett’s test of sphericity, χ^2^(105) = 3312.69, *p <* .001, showed that the 15 GCBS items had adequate common variance for factor analysis. The results of the PAFA revealed three factors with λ *>* 1.0 (6.02, 1.54, and 1.32) and the scree-plot showed a steep cut-off between the primary and secondary factors. Consistent with the EGV1 criterion, the results of parallel analysis indicated that three factors should be extracted (95% CI means = 1.36, 1.28, 1.22). The three extracted factors cumulatively explained 59.1% of the variance. As can be seen in Table 3, six items loaded onto the first factor (loadings = .48-.74), six items loaded onto the second factor (loadings = .47-.69), and 3 items loaded onto the third factor (loadings = .58-.82). Two items (#14 and #15) showed considerable cross-loadings while loading only fairly on their corresponding factors and were, therefore, discarded. The first factor (6 items) was termed “political conspiracies” (Cronbach’s α = .85), the second factor (4 items) was labeled “scientific conspiracies” (Cronbach’s α = .80), and the third factor (3 items) was labeled “extraterrestrial cover-up” (Cronbach α = .77) based on the parent study [22]. In order to test the higher-order factor structure of the measure, we subjected scores on the three subscales to a higher-order PAFA with quartimax rotation. KMO was acceptable (.68) and Bartlett’s test of sphericity was significant, χ^2^(3) = 350.89, *p <* .001. The results revealed a single factor with λ = 1.96, which explained 65.42% of the variance. The loadings were .74 (political conspiracies), .73 (scientific conspiracies), and .61 (extraterrestrial cover-up). These results indicate that the GCBS may have a single higher-order latent factor.

**Table 3:** Descriptive statistics and factor loadings for Generic Conspiracist Beliefs Scale (GCBS) items.

Item	*M (SD)*	Loadings		
		F1	F2	F3
2. The government permits or perpetrates acts of terrorism on its own soil, disguising its involvement.	3.34 (0.98)	.74	.21	.12
3. The government uses people as patsies to hides its involvement in criminal activities.	3.44 (0.95)	.72	.19	.10
1. The government is involved in the murder of innocent citizens and/or well-known public figures, and keeps this a secret.	3.35 (1.06)	.64	.26	.09
4. The power held by heads of state is second to that of small, unknown groups who really control world politics.	3.27 (1.00)	.58	.22	.26
5. A small, secret group of people is responsible for making all major world decisions, such as going to war.	3.31 (1.03)	.56	.20	.32
6. Certain significant events have been the result of the activity of a small group who secretly manipulate world events.	3.31 (1.08)	.48	.23	.35
12. Experiments involving new drugs or technologies are routinely carried out on the public without their knowledge or consent.	3.33 (1.00)	.29	.69	.13
13. Groups of scientists manipulate, fabricate, or suppress evidence in order to deceive the public.	3.28 (1.01)	.32	.65	.13
11. Technology with mind-control capacities is used on people without their knowledge.	3.31 (1.07)	.11	.62	.23
10. The spread of certain viruses and/or diseases is the result of deliberate, concealed efforts of some organizations.	3.26 (1.03)	.15	.56	.34
14. New and advanced technology which would harm current industry is being suppressed.*	3.27 (1.01)	.37	.48	.24
15. A lot of important information is deliberately concealed from the public out of self-interest.*	3.48 (1.02)	.40	.47	.05
8. Evidence of alien contact is being kept from the public.	3.06 (1.09)	.15	.11	.83
7. Secret organizations communicate with extraterrestrials, but keep this fact from the public.	3.08 (1.07)	.21	.19	.63
9. Some UFO sightings and rumors are planned or staged in order to distract the public from real alien contact.	3.08 (1.05)	.13	.24	.58

*Note*. F1 = political conspiracies; F2 = scientific conspiracies; F3 = extraterrestrial cover-up; Items with an asterisk were discarded. The Persian translation is available upon request.

### Inter-Correlations

We calculated bivariate correlations between the scores on the BCTI, CMQ, and GCBS. The correlation coefficients, along with internal consistency coefficients, are presented in Table 4. As can be seen, scores on the BCTI were only weakly correlated with scores on generic measures of conspiracy ideation (.06 < *r* < .14; .002 < *p* < .18). Scores on the CMQ, however, were moderately correlated with GCBS factors (.16 < *r* < .31; *p*s < .001), and the total GCBS scores (*r* = .30, *p* < .001). Factors of the GCBS were highly inter-correlated (.45 < *r* < .54; *p*s < .001).

**Table 4:** Correlation coefficients between conspiracy theories measures.

	1	2	3	4	5	6
1. BCTI	.*85*					
2. CMQ	.14**	.*86*				
3. GCBS-PC	.11*	.31**	.*85*			
4. GCBS-SC	.11*	.25**	.54**	.*80*		
5. GCBS-ET	.06	.16**	.45**	.45**	.*77*	
6. GCBS	.11*	.30**	.81**	.82**	.80**	.*88*

*p < .05

**p < .005

*Note*. BCTI = Belief in Conspiracy Theory Inventory; CMQ = Conspiracy Mentality Questionnaire; GCBS = Generic Conspiracist Beliefs Scale; PC = political conspiracies; SC = scientific conspiracies; ET = extraterrestrial cover-up; internal consistency coefficients are italicized on the diagonal.

### Sex differences

We examined sex differences across all three measures of conspiracist beliefs. In order to correct for multiple comparisons, we corrected our statistical significance to .05/5 = .01. Sex differences in scores on CMQ and GCBS factors were not significant (*p*s > .20). On the other hand, women (*M* = 5.42, *SD* = 1.41) scored significantly higher than men (*M* = 5.06, *SD* = 1.41) on the BCTI, *t*(488) = 2.77, *p* = .006, *d* = 0.25, mean difference = 0.13, 95% CI = 0.10–0.61. This sex difference was small in magnitude.

### Convergent validity

The correlation coefficients between conspiracist belief variables (scores on the BCTI, CMQ, and factors of the GCBS) and other study variables are presented in Table 5. Scores on BCTI were negatively associated with rightist political orientation (*r* = -.12, *p* = .008) and positively correlated with religiosity (*r* = .15, *p* = .001). Because, the relationship between political orientation and conspiracist beliefs could be non-linear (that is, political extremists on both ends endorse conspiracy theories more than moderates [18]), we also examined the correlation between political orientation squared scores and the BCTI scores (*r* = -.11, *p* = .01) which was not substantially different from the correlation between political orientation and BCTI. We also checked quadratic and linear regression lines and they were not substantially different. Therefore, no non-linear quadratic relationship emerged in the current dataset.

**Table 5:** Correlation coefficients between conspiracy theories measures and other study variables.

	BCTI	CMQ	GCBS-PC	GCBS-SC	GCBS-ET	GCBS
Education	-.07	-.03	-.10*	-.08	-.03	-.08
SPQ-B	-.002	.05	.10*	.10*	.17***	.15***
REI analytical-rational	.08	-.09*	-.16***	-.13**	-.17***	-.19***
REI intuitive-experiential	-.05	.17***	.33***	.30***	.23***	.35***
ISBQ Superstitious beliefs	-.03	-.02	.07	.11**	.18***	.15***
ISBQ magical thinking	-.01	.04	.11**	.19***	.17***	.20***
Right-wing political orientation	-.12**	-.07	-.03	-.004	.09*	.03
SRR	.15***	.10*	-.07	-.02	-.04	-.05

*p < .05

**p < .01

***p < .005

*Note*. BCTI = Belief in Conspiracy Theory Inventory; CMQ = Conspiracy Mentality Questionnaire; GCBS = Generic Conspiracist Beliefs Scale; PC = political conspiracies; SC = scientific conspiracies; ET = extraterrestrial cover-up; SPQ-B = Schizotypal Personality Questionnaire-Brief; REI = Rational-Experiential Inventory; ISBQ = Iranian Superstitious Beliefs Questionnaire; SRR = Self-Rating of Religiosity.

Scores on the CMQ were positively correlated with intuitive-experiential information processing style (*r* = .17, *p* < .001) and religiosity (*r* = .10, *p* = .03). These correlations were small in magnitude. Scores on GCBS’s political conspiracies subscale were negatively correlated with education (*r* = -.10, *p* = .02) and analytical-rational information processing style (*r* = -.16, *p* < .001), while positively correlated with schizotypal personality (*r* = .10, *p* = .02), intuitive-experiential information processing style (*r* = .33, *p* < .001), and magical thinking (*r* = .11, *p* = .009).

Scores on GCBS’s scientific conspiracies were positively correlated with schizotypal personality (*r* = .10, *p* = .02), intuitive-experiential information processing style (*r* = .30, *p* < .001), superstitious beliefs (*r* = .11, *p* = .008), and magical thinking (*r* = .19, *p* < .001). In addition, those who scored higher on analytical-rational information processing style reported less political conspiracies (*r* = -.16, *p* < .001). Further, scores on GCBS’s extraterrestrial cover-up factor were positively correlated with schizotypal personality (*r* = .17, *p* < .001), intuitive-experiential information processing style (*r* = .23, *p* < .001), superstitious beliefs (*r* = .18, *p* < .001), magical thinking (*r* = .17, *p* < .001), and rightist political orientation (*r* = .09, *p* = .04). Those who relied on rational processes and analytical thinking reported lower scores on extraterrestrial cover-up conspiracies (*r* = -.17, *p* < .001). Overall GCBS scores were positively correlated with schizotypal personality (*r* = .15, *p* < .001), intuitive-experiential information processing style (*r* = .35, *p* < .001), superstitious beliefs (*r* = .15, *p* < .001), and magical thinking (*r* = .20, *p* < .001). In addition, overall GCBS scores were negatively correlated with analytical-rational information processing style (*r* = -.19, *p* < .001). The correlation coefficient between political orientation squared and GCBS extraterrestrial cover-up scores was close to significance (*r* = .08, *p* = .08) and the quadratic regression line did not diverge from the linear regression line. Therefore, no curvilinear relationship was observed between political orientation and endorsement of extraterrestrial cover-ups.

## Discussion

In the current study, we assessed the psychometric properties of three widely-used measures of conspiracist beliefs, namely the BCTI, CMQ, and GCBS [12,21,22]. Our results indicated difficulties replicating the parent factor structures two of three measures, with only the one-factor model of the CMQ being supported. Beyond factor structures, our results also suggested that evidence of convergent validity was limited, insofar as correlations between scores derived from our translated conspiracist belief measures and additional measures were weak at best. To our knowledge, this is the first empirical study on the psychology of conspiracy theories in Iran and highlights issues with the psychometric properties of existing measures that were originally developed in the West. In what follows, we review measurement qualities of the mentioned measures, discuss their relations to related variables in Iranian culture, and propose future directions in measurement and evaluation of conspiracist beliefs in Iran.

Inspecting the reliability and validity of the Persian BCTI suggests that only 12 items of the original 15 items can be adequately used in Iran. Three items cross-loaded onto a secondary factor and were therefore discarded, though the remaining 12 items loaded onto a primary factor and had adequate internal consistency. More problematically, as we noted above, we encountered broader translational issues with the BCTI–an issue that has previously been highlighted [19]; that is, given that the items of the BCTI were designed to reflect conspiracy theories that were known to Western audiences, they have limited practical use in populations that are not familiar with item content. To take one example, our pilot work indicated that some participants did not know the details about the assassination of Martin Luther King Jr., let alone conspiracy theories surrounding the event. Likewise, although conspiracy theories of the 9/11 terrorist attacks are popular in Iran, the same cannot be said of conspiracy theories concerning the death of Princess Diana. One previous study reported similar issues in a study of French speaking-participants [56], where only 10 of the 15 BCTI items were selected as being recognizable to a French audience. In short, we suggest that the BCTI may show poor item functioning as a result of poor cross-cultural knowledge of content that was designed to be familiar to Western audiences.

On the other hand, we were able to replicate the parent factor structure of the CMQ in our Iranian participants as we expected; that is, all 5 CMQ items loaded onto a single factor with adequate internal consistency, suggesting that scores on this measure are one-dimensional. However, as noted by Swami et al. [19], the CMQ may suffer from issues of construct validity. Most items of the CMQ could be interpreted as factually correct, including in Iran. Indeed, item means on this measure were very high (see Table 2), which is could be interpreted as substantive participant agreement with items that reflect real-world phenomena. To the extent that items were interpreted as being factual, they may be inconsistent with definitions of a conspiracy theory, which require that such theories can be contradicted by the standards of mainstream knowledge. As such, although the CMQ may be factorially valid in Iranian samples, there is possibility that scores on the CMQ suffers from floor effects because of construct validity issues.

Finally, contrary to our hypothesis, we found that scores on the GCBS reduced to a novel three-factor latent structure. Three items that represented Government Malfeasance (GM) and three items that represented Malevolent Global conspiracies (MG) were grouped together under a 6-item factor that we termed “political conspiracies” due to their conceptual government-related malevolence similarity [22]. Three items that Brotherton et al. [22] labeled Personal Well-being (PW) and three items that these authors labeled as Control of Information (CI) were grouped together, which we termed “scientific conspiracies.” Finally, the third factor replicated Brotherton et al.’s [22] Extraterrestrial Cover-up (ET) factor. We suggest that, although the factor structure reported by Brotherton and colleagues [22] was not fully replicated in the present Iranian sample, the alternate three-factor structure is interpretable. Our results also suggest that the three uncovered factors load on to a higher-order dimension, although it will be important to replicate this finding using CFA. In short, the factor structure of the GCBS in Iran does not mirror the parent factor structure reported by Brotherton et al. [22], nor does it mirror alternate factor structures that have been proposed for this measure [19].

Beyond factor structures, our results also suggested that scores on all three measures displayed relatively weak evidence of convergent validity. More specifically, scores on the BCTI were only significantly correlated with greater religiosity and more leftist political orientation, although the effects were weak. Likewise, scores on the CMQ were only significantly correlated with greater intuitive thinking, but again the effect was weak. Scores on the GCBS fared better in terms of convergent validity, as we found significant associations between some GCBS factors and education, schizotypal personality, information processing styles, culture-specific superstitious beliefs, and rightist political ideology. However, it should be noted that the pattern of correlations was not consistent across GCBS factors, and most significant correlations were weak at best. We also found that Iranian women scored higher on the BCTI, which is consistent with recent research indicating that Iranian women score higher on measures of superstition and magical thinking [36], yet this gender effect should be treated with caution until further replicated in future studies. A further issue of concern was the fact that, although scores on the three measures of conspiracist beliefs were significantly inter-correlated, the effect sizes of the associations were weak-to-moderate indicating poor evidence of convergent validity. This suggests that the three measures included in the present study may be tapping distinct constructs, at least in the present Iranian sample.

Two related explanations lend themselves in relation to the poor evidence of convergent validity. The first is that the three measures of conspiracist ideation are not adequately tapping the latent construct in the Iranian context, an issue we return to below. The second is that antecedents of conspiracist beliefs that have been reported in Western contexts may not be reliable correlates in Iran, possibly because of culture-specific issues. For example, Pipes [28] has suggested that the spread of conspiracy theories in the Middle East could be linked with the fortunes of political Islam, the contested nature of European thought, the plethora of actual conspiracies, and the specific nature of Middle Eastern politics, particularly pan-movements and autocracy. To this list, one might also add the political destabilization that has occurred in the region as a result of Western military intervention, as well as the role of the state in narrating and spreading conspiracy theories [29–30]. A further distinctive issue concerning conspiracy theories in the Middle East, including in Iran, is their widespread use by political and religious leaders as a means of mobilizing populist support [27,57]. In such a scenario, the conceptual meaning and function of conspiracist beliefs in Iran may be different to that in the West. As a result, correlates that were implicated in conspiracist beliefs in the West may not be reliable antecedents in Iran specifically or the Middle East more broadly.

Although the present work has strengths (e.g., an adequately powered sample size, non-Western culture, non-student sample), limitations are worth noting. First, we did not include other measures of conspiracy theories such as the Conspiracy Theory Belief Scale [58] or the One-Item Conspiracy Measure [56]. As mentioned, we aimed to include measures that fared psychometrically well and that have been widely-used in previous research. Yet, it is possible that these alternative measures may show better psychometric properties in non-Western societies. Therefore, it is recommended for future research to examine the factorial validity of the Persian translations of other existing measures of conspiracy ideation. Second, we did not examine the factorial validity of the measures using CFA. It is, therefore, recommended for future research to examine the latent structure of these measures by the means of comparing models developed in the parent studies and the models developed in the current exploratory analyses. Third, we did not assess the temporal stability of the three measures. As conspiracy beliefs are trait-like constructs that are not easily altered in short time intervals (see [59]), it is recommended for future research to examine test-retest reliability of these measures. Finally, future work could assess convergent validity through additional variables that may be more context-specific, such as variables that are derived from social identity theory [60].

In summary, the present results suggest that scales developed to measure conspiracist beliefs in the West may not be entirely suitable for use in Iran. It is reasonable to assume–given the issues with factorial and construct validity discussed above–that the three measures included in the present study do not adequately assess the nature, meaning, and function of conspiracy theories in the Iranian context. Developing a context-specific measure of conspiracist beliefs would seem to be the best way forward. In particular, it would be useful to begin with qualitative research that seeks to more concretely understand the nature and function of conspiracy theories in Iran (or the Middle East), based on existing knowledge [27,28,30,61]. Such research could then form the basis for developing a new measure of generic conspiracist beliefs that is suitable for with Iranian participants. Alternatively, it should be possible to develop a measure of conspiracist beliefs that more accurately reflects conspiracy theories that are prevalent in Iran; that is, such a measure would be context- and culture-specific, with item content more usefully reflecting local knowledge and beliefs. Until such measures are available, scholars intending to assess conspiracist beliefs in Iran could use the 12-item BCTI or the 13-item GCBS, although we strongly advise scholars to assess factorial validity in their own samples. Use of the CMQ, on the other hand, should perhaps be limited, given known issues of construct validity and the ceiling effects reported in the present work.

## Appendix 1

1. A powerful and secretive group, known as New World Order, is planning to eventually rule the world through an autonomous world government, which would replace sovereign government.
2. SARS (a respiratory virus whose outbreak in 2003 affected thousands of individuals) was produced under laboratory conditions as a biological weapon.
3. The US government had foreknowledge about the Japanese attack on Pearl Harbor (in 1941), but allowed the attack to take place so as to be able to enter the Second World War.
4. US agencies intentionally created the AIDS epidemic and administered it to Black and gay men in the 1970s.
5. The assassination of Martin Luther King, Jr. (leader of the civil rights movement in the US, assassinated in 1968), was the result of an organized conspiracy by US government agencies such as CIA and FBI.
6. The Apollo moon landings (i.e., landing the first humans on the Moon by NASA in 1960s) never happened and were staged in a Hollywood film studio.
7. Area 51 in Nevada, US, is a secretive military base that contains hidden alien spacecraft and/or alien bodies.
8. The US government allowed the 9/11 attacks to take place so that it would have an excuse to achieve foreign (e.g., wars in Afghanistan and Iraq) and domestic (e.g., attacks on civil liberties) goals that had been determined prior to the attacks.
9. The assassination of John F. Kennedy (American President, in office from 1961 until his assassination in 1963) was not committed by the lone gunman, Lee Harvey Oswald, but was rather a detailed, organized conspiracy to kill the US President.
10. In July 1947, the US military recovered the wreckage of an alien craft from Roswell, New Mexico, and covered up the fact.
11. The death of Princess Diana (a member of the British royal family) was not an accident, but rather an organized assassination by members of the British royal family who disliked her.
12. The Oklahoma City bombers (in 1995), Timothy McVeigh and Terry Nichols, did not act alone, but rather received assistance from neo-Nazi groups.
13. The Coca Cola company intentionally changed to an inferior formula with the intent of driving up demand for their classic product, later reintroducing it for their financial gain.
14. Special interest groups are suppressing, or have suppressed in the past, technologies that could provide energy at reduced cost or reduced pollution output.
15. Government agencies in the UK are involved in the distribution of illegal drugs to ethnic minorities.

## Supporting information

S1 Data CT_PLOS_ONE_2.sav.zip. (ZIP)
